# Assisted Reproduction: What factors interfere in the professional's decisions? Are single women an issue?

**DOI:** 10.1186/1472-6874-11-21

**Published:** 2011-05-31

**Authors:** Suzana Záchia, Daniela Knauth, José R Goldim, Juliana R Chachamovich, Eduardo Chachamovich, Ana H Paz, Ricardo Felberbaum, PierGiorgio Crosignani, Basil C Tarlatzis, Eduardo P Passos

**Affiliations:** 1 Post-Graduate Program in Medicine, Assisted Reproduction Service, Gynecology and Obstetrics Department, Hospital de Clínicas de Porto Alegre 90035-903, Porto Alegre, Brazil; 2 Social Medicine Department, Federal University of Rio Grande do Sul, Porto Alegre, Brazil; 3 Research And Post-Graduation Group, Hospital de Clínicas de Porto Alegre, Porto Alegre, Brazil; 4 Post-Graduate Program in Psychiatry, Federal University of Rio Grande do Sul, Porto Alegre, Brazil; 5 Department of Obstetrics and Gynecology, Clinic Kempten-Oberallgaeu, Kempten, Germany; 6 Department of Obstetrics and Gynaecology, Istituto Luigi Mangiagalli, University of Milan, Milan, Italy; 7 Unit for Human Reproduction, 1st Department of Obstetrics and Gynaecology, Aristotle University of Thessaloniki, Papageorgiou General Hospital, Nea Efkarpia Peripheral Road, Thessaloniki 54603, Greece

## Abstract

**Background:**

With the development of medical technology, many countries around the world have been implementing ethical guidelines and laws regarding Medically Assisted Reproduction (MAR). A physician's reproductive decisions are not solely based on technical criteria but are also influenced by society values. Therefore, the aim of this study was to analyze the factors prioritized by MAR professionals when deciding on whether to accept to perform assisted reproduction and to show any existing cultural differences.

**Methods:**

Cross-sectional study involving 224 healthcare professionals working with assisted reproduction in Brazil, Italy, Germany and Greece. Instrument used for data collection: a questionnaire, followed by the description of four special MAR cases (a single woman, a lesbian couple, an HIV discordant couple and gender selection) which included case-specific questions regarding the professionals' decision on whether to perform the requested procedure as well as the following factors: socio-demographic variables, moral and legal values as well as the technical aspects which influence decision-making.

**Results:**

Only the case involving a single woman who wishes to have a child (without the intention of having a partner in the future) demonstrated significant differences. Therefore, the study was driven towards the results of this case specifically. The analyses we performed demonstrated that professionals holding a Master's Degree, those younger in age, female professionals, those having worked for less time in reproduction, those in private clinics and Brazilian health professionals all had a greater tendency to perform the procedure in that case. A multivariate analysis demonstrated that the reasons for the professional's decision to perform the procedure were the woman's right to gestate and the duty of MAR professionals to help her. The professionals who decided not to perform the procedure identified the woman's marital status and the child's right to a father as the reason to withhold treatment.

**Conclusion:**

The study indicates differences among countries in the evaluation of the single woman case. It also discloses the undervaluation of bioethics committees and the need for a greater participation of healthcare professionals in debates on assisted reproduction laws.

## Background

The impossibility of bearing children may result in a series of negative feelings, such as sadness, guilt and social isolation. It may also be deemed one of life's crises in which the stability of a couple's relationship may be at stake [1]. Yet over the last decades, the ever-growing high technology in this field has been allowing women and couples to fulfill their wishes of pregnancy.

Since the emergence of in vitro fertilization (IVF) in 1978, the continuing development of assisted reproduction technologies MAR has led to complex ethical, legal and social issues related to their applications [2]. Insemination with donor semen and the new IVF-based techniques have detached conception from sexual intercourse thus enabling the involvement of a third party in the reproduction process, and have thereby challenged the traditional family identity [2].

According to Fasouliotis and Schenker most professional teams in the various countries they studied in 1999 recommended that MAR be restricted to heterosexual couples who are legally married or at least living in a stable relationship. In Europe, this practice was offered in countries like Belarus, Italy and Spain and it was often the case where 2 years of cohabitation fulfilled the marital requirement to have MAR performed (i.e. France). Whereas in Iran, Saudi Arabia and Jordan, where religion significantly influences social life, marriage was usually the requirement. Yet they found that other Asian countries such as India and China also allowed these procedures to cohabiting couples [3].

However, in modern days there are specific situations in which health teams must discuss and review the possibility of using MAR especially given the ethical ambiguities they generate. Some of these are: gender selection, embryo cryo-preservation, female homosexual couples who wish to use donor semen, HIV serodiscordant couples who wish to undergo insemination, among others [3-6]. In order to attempt to meet these needs, these couples search for specialized centers to request that trained professionals help them by doing the procedure and it is precisely in these situations that values, culture, knowledge and experience assume greater importance in the professionals' final decisions.

Thus, considering the growing importance of this matter, since 1990 many countries have been setting out to establish ethical guidelines and laws for reproductive technologies [7].

Yet in the Brazilian context there is no specific legislation ruling the procedure and therefore MAR is basically offered to heterosexual couples who are in stable relationships. In 1992, however, the Brazilian Medical Board enacted a resolution [8] aimed at orienting MAR professionals, which is currently used merely as a guideline. In Brazil, this resolution states that the use of assisted reproduction techniques is conditioned to the presence of infertility. In Germany and Italy, insemination of a single woman is not allowed and in Greece, single women may have children through assisted reproduction yet a notarial deed is required. It is precisely due to this lack of official laws monitoring the use of MAR that diversity-related controversies as those mentioned earlier arise.

Also, given that the use of this technology involves high costs [9] much of the use of MAR in Brazil is made under private care, which could explain private clinics acting more independently.

Therefore, scientific research on attitudes and behaviors regarding reproduction has become important and should be expanded so as to better understand the opinions held by professionals in this field and assist in the development of training programs for these professionals. The present study aimed at verifying whether there are differences among countries in the way their professionals analyze and decide on controversial cases of assisted reproduction.

## Methods

### Study population

Between July, 2003 and July, 2005, a cross-sectional study was carried out with a sample of healthcare professionals working in assisted reproduction in Brazil and in three countries of the European Community (Germany, Greece and Italy). In order to ensure that the healthcare professionals invited to join were indeed qualified in MAR, we contacted 544 professionals who, in 2003, were members of either of the following well-established societies of assisted reproduction: the European Society of Human Reproduction and Embryology (ESHRE) or the Brazilian Society of Assisted Reproduction (SBRA). Out of the 544 registered professionals we were able to have access to 327 e-mail addresses that had been provided accurately in said societies.

### Data collection

For data collection purposes, a program was designed to incorporate two systems. One was a safe administrative interface granting restricted access to authorized persons only, who, in turn, were able to manage information on the database and export items to an SPSS (*Statistical Package for the Social Sciences*) data file for further analyses. The other one was an online version capable of verifying typed information. This version contained a socio-demographic questionnaire, which once completed gave access to the description of four assisted reproduction cases and related questions (in English for the Europeans and in Portuguese for the Brazilians).

Each of the 327 professionals was sent an e-mail invitation written in his or her country's official language informing of the study. The professionals accepted the invitation by clicking on an "accept" icon which led them directly to the site (online version) and granted access to the questionnaire and cases available therein. Once they had completed all items, their answers were automatically submitted to the restricted area of the database yet detached from any personal identification so as to assure confidentiality. Completion of the questionnaire was taken as implied consent, that is, by sending their answers, the participants fully accepted the terms of the study.

The data gathered were the professionals' socio-demographic information and their answers to questions regarding their professional and personal interpretation of those four true cases of assisted reproduction. These four cases reached the university hospital team and, due to their complexity, had to be discussed by a committee so as to decide whether a procedure could be performed or not in each case. A pilot study involving a small group of experienced MAR professionals was carried out so as to identify the main factors they would take into account and elements that would underpin their decisions if they were asked to perform MAR in such scenarios. These professionals were asked to write their opinions in full and it was based on their answers that categories were generated and a questionnaire was created for our study. The cases had already been subjected to the evaluation of the Bioethics Committee of a university hospital. The research project was approved by the Research Ethics Committee under number GPPG-02405. All regulatory aspects were fully addressed, namely the Helsinki Declaration.

Briefly, the cases were described as follows:

Case 1) A single middle-class woman with no intention of having a male partner in the future acquires 5 samples of semen in a commercial cryobank. She has had 3 unsuccessful inseminations and thus comes to another MAR Centre requesting a new attempt.

Case 2) A lesbian couple wishing to have a child requests that the clinician of an Assisted Reproduction Service obtain an oocyte from one of the partners to be fertilised with semen from a sperm bank. The embryo should then be transferred to the other partner who will act as a surrogate, so that both can participate actively in the process (one genetically and the other one by carrying the baby).

Case 3) A non-infertile couple requests a homologous insemination because the woman is HIV positive. The purpose of the request is to protect the husband from being exposed if they are to have sexual intercourse without a condom. In the interview with the MAR team, the couple says that if they get a negative answer from the centre they will try to have a baby anyway by having sexual intercourse without protection.

Case 4) A heterosexual couple, who has two children, goes to a Human Reproduction Centre because they wish to have another child, yet due to a tubal problem, she is unable to have her ovum fertilized naturally. Considering their request involves a technical procedure and they already have two boys, they would only like female embryos to be implanted.

The factors examined were the professionals' socio-demographic variables, their moral (fairness, respect for people, beneficence) and legal values as well as the technical aspects that contributed to their final decisions. Based on the answers provided by the professionals in the pilot study, the researchers selected the most significant factors described as having influenced the professionals' decision-making process and created the questionnaire. The factors selected were: respect for a child's right to a father; the right to choose to gestate; the professional duty to help the patient/couple/person having the procedure; the marital status of the patient (one patient does not want to have a partner in the future); and socio-economical level (which would enable the patient to provide the child with quality of life). The questionnaire then required that the respondents define whether their final decision was further influenced by what they understood as being their technical, moral (fairness, respect for people and beneficence), or legal values. The outcome measured was whether or not these professionals accepted to perform the procedure. The quantitative variables were described by means and standard deviations or medians, and the 25-75 percentiles and the qualitative variables (country, gender, profession, marital status, having children or not, educational level and the decision to perform or not the procedure) were described as absolute and relative frequencies.

## Statistical Analysis

The data were compared in bivariate analyses using the Chi-square or Fisher tests to evaluate the association between the qualitative variables. The Analysis of Variance or the Kruskal-Wallis Test was used to compare the age and the length of time the professionals had been working in reproduction relative to the countries. To assess these same variables with regard to the decision to perform the procedure or not, the Student's t Test or the Mann-Whitney test was used. In the multivariate analysis, the logistic regression technique was used. The adjusted odds ratio and its confidence interval were calculated to measure effect size. The significance level adopted in this study was 5% and the SPSS version 10.0 was utilized for the statistical analysis.

## Results

A total of 327 professionals received the e-mail invitation out of which 16 (4.9%) did not accept to participate in the study, 87 (26.6%) only accessed the site, but did not answer the questionnaire, and 224 (68.5%) fully accepted to participate in the study. The sample of the study thus consisted of 224 health professionals: 51.1% (n = 115) Brazilians; 22.2% (n = 50) Germans; 17.7% (n = 40) Italians and 8.4% (n = 19) Greeks. The socio-demographic characteristics of the professionals who participated in the study are: most were male (71.1%); physicians (84.0%) or biologists (12.7%); living with a partner (83.5%) and had children of their own (76.0%). These variables were not statistically significant when comparing across countries (table 1).

**Table 1:** Characteristics of the Sample According to country of Employment

Variables	According to Country of Employment				P
		
	Brazil	Germany	Italy	Greece	
**Sex-n (%)**					
Male	77 (67.0)	38 (76.0)	30 (75.0)	15 (78,9)	0.491***
Female	38 (33.0)	12 (24.0)	10 (25.0)	4 (21.1)	
Age* - mean ± sd	43.0^b ^± 9.3	45.0^ab ^± 7.4	47.5^a ^± 9.8	42.4^ab ^± 7.8	0.036****
**Children-n (%)**					
Yes	86 (74.8)	40 (80.0)	31 (77.5)	13 (68.4)	0.759***
No	29 (25.2)	10 (20.0)	9 (22.5)	6 (31.6)	
**Living with partner-n (%)**					
Yes	92 (80.0)	43 (86.0)	37 (92.5)	15 (78.9)	0.274***
No	23 (20.0)	7 (14.0)	3 (7.5)	4 (21.1)	
**Profession-n (%)**					
Medicine	93 (86.9)	39 (84.8)	34 (85.0)	12 (66.7)	0.708***
Nursing	1 (0.9)	0 (0.0)	0 (0.0)	0 (0.0)	
Psychology	2 (1.)	1 (2.2)	1 (2.5)	1 (5.6)	
Biology	11 (10.3)	6 (13.0)	5 (12.5)	5 (27.8)	
Time working in the field** median (P 25-P 75)	10.0^b ^(5.0-15.0)	13.0^ab ^(7.8-19.0)	15.0^a ^(10.0-20.0)	10.0^b ^(5.0-14.0)	0.001*****
**Public Center-n (%)**	35 (30.4)	27 (54.0)	24 (60.0)	7 (36.8)	0.002***
**Private Center-n (%)**	98 (85.2)	26 (52.0)	27 (67.5)	13 (68.4)	<0.001***

* Equal characteristics do not differ on the Tukey test

** Equal characteristics do not differ on the Dunn test

*** Value obtained using the Chi-square test

**** Value obtained by the analysis of variance

***** Value obtained by the Kruskal-Wallis test

The professionals' mean age was 44.2 years (sd = 9) whereas the Italian professionals had a mean age greater than that of the Brazilians (p = 0.036). The median length of time working with MAR was 12 years; the Italians having worked in the field for a longer time when compared to the Greeks and to the Brazilians (p < 0.001).

Over 75% of the professionals, regardless of country, had graduated in the country where they worked (p < 0.001) and over 50% also had post-graduate degrees from the same country where they worked (p < 0.001). When analyzing the post-graduation level of the participants, it was found that the Italians were associated with specializations; the Brazilians with Master's Degrees; the Greeks with PhDs; and the Germans with Post-Docs (p < 0.001). Most of the Italians and Germans worked in public centers (p = 0.002) while most of the Brazilians and Greeks worked in private ones (p < 0.001).

In the evaluation of the four cases relative to socio-demographic characteristics and countries, the only statistically significant association (p < 0.001) regarding the professionals' decision to perform the assisted reproduction procedure (or not) occurred in the case of the single woman with no partner (case 1). Therefore, the statistical analyses of this study were performed based exclusively on the professionals' answers regarding that case.

The comparisons between the variables studied and the professionals' decision on whether to perform the procedure in that case identified that younger, female professionals, those holding a Master's Degree, those in private clinics, those having practiced for a shorter time in the field of reproduction and Brazilian professionals were all more likely to perform the procedure (table 2).

**Table 2:** Comparison Between the Studied variables and the decision to perform or not the procedure in case 1 (a single woman with no intention of having a male partner in the future)

Variables	Perform the procedure in case 1?		P
		
	Yes	No	
**Sex-n (%)**			
Male	61 (38.9)	96 (61.1)	0.004*
Female	38 (61.3)	24 (38.7)	
**Age - mean ± sd**	42.2 ± 8.6	45.7 ± 9.2	0.005**
**Country of Employment - n (%)**			
Brazil	66 (59.5)	45 (40.5)	< 0.001*
Germany	14 (28.6)	35 (71.4)	
Italy	8 (20.5)	31 (79.5)	
Greece	10 (52.6)	9 (47.4)	
**Children-n (%)**			
Yes	75 (45.5)	90 (54.5)	1.000*
No	24 (44.4)	30 (55.6)	
**Living with Partner -n (%)**			
Yes	80 (43.5)	104 (56.5)	0.321*
No	19 (54.3)	16 (45.7)	
**Profession-n (%)**			
Medicine	73 (42.0)	101 (58.0)	0.228*
Nursing	2 (100.0)	0 (0.0)	
Psychology	2 (40.0)	3 (60.0)	
Biology	14 (56.0)	11 (44.0)	
**Graduate Studies - Level -n (%)**			
Specialization	39 (44.3)	49 (55.7)	0.040*
Master's Degree	18 (69.2)	8 (30.8)	
PhD	19 (42.2)	26 (57.8)	
Post-Doc	12 (33.3)	24 (66.7)	
**Time working in the field - median (P25-P75)**	10.0 (5.0 - 15.0)	13.0 (7.0 - 20.0)	0.010***
**Public Center - n (%)**	27 (29.7)	64 (70.3)	< 0.001*
**Private Center - n (%)**	82 (51.6)	77 (48.4)	0.003*

* Value obtained by the Chi-square test

** Value obtained by the Student-t Test

*** Value obtained by the Mann-Whitney Test

**** Value obtained by the Fisher Exact Test

The statistically relevant aspects taken into account by the professionals who accepted to perform the procedure in that case were: the socio-economic level of the patient, the right to choose to gestate, the professional's duty to help the patient by performing the procedure as well as technical aspects. Conversely, the statistically relevant aspects most considered by those who chose not to perform it were the patient's marital status and the child's right to a father (table 3).

**Table 3:** Evaluation of aspects relevant to the decision of performing or not the procedure in case 1 (a single woman with no intention of having a male partner in the future)

Aspects	Perform the procedure in case 1?		P*
		
	Yes n (%)	No n (%)	
**Marital Status of the Patient**	19 (21.1)	71 (78.9)	**< 0.001**
**Socio-economical Level**	54 (58.1)	39 (41.9)	**0.002**
**Right to choose to gestate**	89 (59.7)	60 (40.3)	**< 0.001**
**Child's right to a father**	42 (29.8)	99 (70.2)	**< 0.001**
**Professional's duty to help the patient have the procedure**	75 (64.1)	42 (35.9)	**< 0.001**
**Need for an evaluation of the case by a bioethics committee**	43 (38.4)	69 (61.6)	0.762
**Technical Aspects**	57 (75.0)	19 (25.0)	**< 0.001**
**Moral Aspects**	60 (43.2)	79 (56.8)	0.607
**Legal Aspects**	42 (38.9)	66 (61.1)	0.849

* Value obtained by the Chi-square test with Yates correction

For the Italians who would not perform the procedure in case 1, legal aspects were considered highly relevant (p = 0.037). For the remaining countries, there was no association between legal aspects and the decision not to perform the procedure: Brazil (p = 1.000), Germany (p = 0.299) and Greece (p = 1.000).

The logistic regression model showed that the professionals who considered the right to gestate as relevant had a 3.88-fold greater chance of performing the procedure in the single woman case than did those who did not offer this justification (OR = 3.88; 95% CI = 1.11 - 13.49). Those who regarded the professionals' duty to help the patient as relevant had a 2.88-fold greater probability of performing it than did those who did not find this aspect to be pertinent (OR = 2.88; 95%CI = 1.06 - 7.83). In contrast, those professionals indicating the patient's marital status as relevant were 87% less likely to perform the procedure (OR = 0.13; 95%CI = 0.04 - 0.37). Likewise, those professionals considering the child's right to a father as relevant had a 75% (OR = 0.25; 95%CI = 0.09 - 0.72) reduced likelihood of performing the procedure (table 4).

**Table 4:** Multivariate analysis of the predicting variables for the decision to perform or not the procedure in case 1 (a single woman with no intention of having a male partner in the future)

Variables	Adjusted OR	95%CI	P
			
**Sex**			
Male	1.00		
Female	2.29	(0.77 - 6.80)	0.135
**Age**	0.98	(0.93 - 1.04)	0.521
**Country of Employment**			
Brazil	1.00		
Germany	0.41	(0.12 - 1.47)	0.172
Italy	0.40	(0.10 - 1.63)	0.204
Greece	1.68	(0.31 - 9.07)	0.544
**Profession**			
Medicine	0.40	(0.11 - 1.47)	0.169
Others	1.00		
**Graduate Studies - Level**			
Specialization	1.00		
Others	1.88	(0.66 - 5.27)	0.233
**Public Center**			
Yes	1.00		
No	1.55	(0.60 - 3.98)	0.362
**Marital Status of the Patient**			
Yes	0.13	(0.04 - 0.37)	**< 0.001**
No	1.00		
**Socio-Economical Level**			
Yes	2.66	(0.98 - 7.28)	0.056
No	1.00		
**Right to choose to gestate**			
Yes	3.88	(1.11 - 13.49)	**0.033**
No	1.00		
**Child's right to a father**			
Yes	0.25	(0.09 - 0.72)	**0.010**
No	1.00		
**Professional's duty to help the patient have the procedure**			
Yes	2.88	(1.06 - 7.83)	**0.039**
No	1.00		
**Need for an evaluation of the case by a bioethics committee**			
Yes	0.54	(0.20 - 1.47)	0.224
No	1.00		
**Technical Aspects**			
Yes	1.97	(0.70 - 5.56)	0.198
No	1.00		
**Legal Aspects**			
Yes	1.54	(0.59 - 3.99)	0.374
No	1.00		

In case 2, which relates to a lesbian couple who both wish to participate actively in the pregnancy process, 76.4% of the respondents opted against the procedure, and 23.6% were in favor of performing it. Those who opted against the procedure marked the following factors to justify their decision: 81.2% referred to the child's conflict regarding the true identity of the father, and 72.1% were concerned about the prejudice the child may face in the future.

On the other hand, in case 3, where a serodiscordant couple requested artificial insemination, 63% said they would perform the procedure whereas 37% would not. The most relevant factors chosen to justify performing the procedure were the following: the wish to gestate (86%), the couple's right to the best treatment available (98.5%), risk prevention for the husband (90%), technical feasibility (53%) and technical aspects (71%).

In case 4, which concerned gender selection, 76.4% of the professionals would perform the procedure and 23.6% would not. Those who would not do it justified their decision as being related to legal impediments (74.8%) and moral aspects (64.5%).

## Discussion

The present study identified the variables which interfered cross-culturally in the decision-making process of healthcare professionals who work with assisted reproduction. Most of the participants were physicians (84%).

It was believed that some of the professionals' socio-demographic factors such as sexual inclination, having children (or not), and living with a partner (or not) could interfere with their decisions regarding assisted reproduction. However, no statistically significant association was found between these aspects and the final outcome in the case of the single woman analyzed herein.

On the other hand, the age of the professionals and the length of time they had been employing MAR were found to be statistically significant. The Italians' older age, which corresponded to a longer time practicing in the field, and thus their consequent greater experience in assisted reproduction, may explain the value they attach to legal aspects when justifying why they would not perform the procedure in the case studied. The fact that the 3 European countries precede Brazil in matters of assisted reproduction [10] also grants them a further consolidated legislation, as well as a possible greater commitment to such regulations.

Another fact which, to some extent, reflects the more recent nature of assisted reproduction in Brazil is that most of the Brazilian professionals interviewed (85.2%) work in private centers. The incorporation of new reproductive technologies into the public health system in Brazil, as in other Latin American countries, is still incipient [9,11,12].

Performing non-standard assisted reproduction procedures (whereas standard is an infertile heterosexual couple's request), here represented by a single woman's request for insemination, was better accepted by those who had been in the field for less time and by those who practiced in private centers. The Brazilians and the women also tended to be more tolerant towards this case, accepting to perform the procedure more easily. The fact that the case relates to a woman's wish might be the reason why female professionals (61.3%) demonstrated a greater degree of tolerance; they might better identify themselves with the situation. Similarly, one may also assume that younger professionals have different values as compared to older ones especially considering the progress of technology and modern life changes, namely concerning novel family compositions [13]. On the other hand, the fact that Brazilians (60%) showed more tolerance could lead to the assumption that the culture in which they live influences with the decisions they make. The widespread occurrence of Brazilian families "headed by women" [14] and the strong association existing between reproduction and woman in Brazil [15] cause the single-woman-with-no-partner situation not to be perceived as unusual, as might be the case in some European countries.

Legal aspects, though reported as conflicting, were not insurmountable obstacles to MAR procedures. Even in Germany and Italy, where it is illegal to inseminate single women, almost one fourth of the professionals (24.4%) accepted to perform the procedure, and those who did not, did not always justify the decision on legal grounds.

In Brazil, there is no legislation ruling assisted reproduction in Brazil. Yet there is a resolution by the Federal Medical Board which orientates physicians as to the proper conduct when faced with problems concerning MAR. The resolution does not mention single women or homosexual couples specifically and endorses the use of assisted reproduction techniques solely in cases involving infertility [8]

However, the Brazilians who accepted to inseminate the woman in case 1 clearly disregarded said formal impediment and the fact that the patient was not infertile. This non-compliance with the formal resolution may be related to different factors, one of which is the fact that the professionals are not held liable or punishable by this resolution because it does not have as much power as a law. In addition, the fact that most of the Brazilian professionals work in private centers might enable them to act more independently. Furthermore, they might not be fully aware of the details of the resolution, given that it stipulates that the beneficiary must have been diagnosed with infertility and the case at hand did not mention whether the woman had been diagnosed or not, only that she wished to be a mother without having to commit to a partner.

A bill currently being examined by the Brazilian senate states that assisted reproduction techniques must only be granted permission in cases of infertility and in those concerning the prevention of sex-related genetic disorders. For such techniques to be conceded there must be a medical indication therefor and voluntary and informed consent is mandatory for both beneficiaries when the woman receiving treatment is married or in a domestic partnership [15,16].

In the multivariate analysis, two aspects emerged as relevant for the professionals' decision to carry out the insemination of the single woman without a partner: the patient's right to gestate and the professional's duty to help the patient by performing the procedure.

Assisted reproduction techniques have fulfilled the reproduction wishes of a significant number of couples. However, it has been observed that individual realization is also being sought by means of this technology, a phenomenon which has lead professionals to express caution about the procedure in such cases. The impossibility of procreating is indeed an obstacle for a woman's plans, and the recourse to assisted reproduction can be commonly observed within cultural contexts marked by individualism and autonomy [15]. Moreover, the incidence of births to single or unmarried persons has grown worldwide, including among never-married, college-educated, professional women.

The professionals who were favorable to performing the procedure in a single woman valued precisely this individual right to motherhood. However, one must bear in mind that most of these professionals work in private centers, which means they cater for a higher income population. Thus the question remains whether this autonomy and freedom would be equally valued in the case of a woman in a poor setting [15].

Also related to the right to gestate is the concept that professionals have the duty to help their patients assert such a right. That is, the professionals who choose to perform the requested insemination play the role of executors or facilitators of a wish [17]

This perception of the professional's duty to help seems to be related to two main factors: on the one hand, there is the relative "easiness" of the requested technique (insemination), which within assisted reproduction procedures may be regarded as the least complex one [18]; on the other hand, there is the professional-client relationship, in which the professionals linked to the private sector often feel pressured to meet their patients' demands, especially given the growing competition in health care.

In opposition to this concept of safeguarding women's reproductive rights there is a group of professionals (54.8%) who justify declining to perform the procedure precisely on the grounds of the individualistic nature of the request. They are driven by the patient's marital status and the child's right to a father. In light of current reproductive technologies, women who wish to gestate no longer depend on sexual intercourse for such, as they may have access to sperm banks in order to reach that goal. However, even if their technical "problem" may be easily overcome through the use of MAR, which also offers the woman and child some added advantages such as lowered risk of sexually-transmitted infections [19], the social and cultural issues still remain.

One of such issues refers to the identity of the sperm donor [20,21]. Some authors are concerned that keeping it a secret may have negative effects on the family relationship and on the child. A growing number of programs in several countries currently have donors in their records who allow their identities to be released. In the first *Identity*-Release^® ^sperm bank in the United States of America -The Sperm Bank of *California *(TSBC), the donors may choose to release their data, thereby allowing the interested offspring, at the age of 18 or older, to learn their identity. This is a very popular option, with almost 80% of their recipients seeking this type of donor [21]. The option of revealing donor identity has also come up in other countries in which there was a formal recognition of the child's rights concerning his/her genetic origins. The donor's option of having his identity revealed was first legislated in Sweden, followed by Austria, Switzerland, Australia and, more recently, the Netherlands. A potential benefit deriving from this disclosure is that it would help avoid a sense of futility regarding the child's origins as well as the possible negative repercussions caused by the lack of this information. However, it is not yet clear whether these parents, knowing the donor's identity, would in fact reveal it to their children [21].

European studies have shown that the decision for revealing varies among families and among countries. Only a few parents have told their pre-adolescent children about their origins. In the UK, Golombok et al found that only 5% of parents had told their near-adolescent children about their conception origins [22]. Additional studies in Europe showed similar trends among families with pre-adolescents; none of the Italian parents told, very few (4%) Spanish ones did; and 23% of Dutch parents did [21]. There is no consensus in the literature regarding the positive impact of revealing the use of MAR to ones' children. The available data from studies performed in different countries on the revelation of having used MAR indicate that parents have a strong tendency to omit this information from their children [23-25].

Women who do not live with a partner and thus did not face the issue of male infertility are likely to better accept the idea of revealing the donor's identity. Scheib *et al *have shown revelation rates close to 100% in these cases and state that little is known about the children's reactions or their late feelings about their origins [21].

The absence of a male parent in the lesbian family structure leaves these couples no choice but to be open with their children about the use of a donor. The fact that this person is not missing in a heterosexual family gives these parents the opportunity to decide whether or not to keep the artificial means of conception secret from their children [26].

Unlike heterosexual couples, single mothers and lesbian couples must explain the absence of a father to their children and thus are more likely to disclose information about the conception to their children [27,28]. Due to the lack of research, very little is known about how single women confront this issue when their children begin inquiring about their father [29].

Even in families whose genetic parents have made use of reproductive technology there remains a concern regarding revealing to the children that they were generated with the use of reproductive technology. In such cases, genetic fatherhood is not questioned, but having needed medical help to generate the children is. A growing number of studies into the development of and future repercussions for children born through assisted reproduction can be found in the literature [20,21,30].

The use of assisted reproduction technology may have consequences which surpass the mere use of the technique for the fulfillment of a woman's desire and may have repercussions for her offspring. Those professionals who decided against the procedure in this case considered that the child's right to a father was a powerful argument.

The reluctance of some professionals to offer assisted reproduction technologies to a single woman with no intention of having a partner in the future could also be related to the fact that they believe that the presence of the father is fundamental for the psychological development of the child. Traditionally, in the western culture, the father is seen as the responsible authority for setting limits, while the mother is to be in charge of the emotional and affective aspects of family life [31]. The healthcare professionals who opposed to insemination in this case might not only be concerned with the absence of the father figure in the child's life but also with the importance of this woman's social relationships. According to Boivin, the quality of the social support fostered by this fatherless child's family is very important for his/her development, and the interaction with other adult models must be assured [19].

The main limitation of this study is the tool used for data collection: the internet. Many professionals may have disregarded the invitation to participate in this study because they feared computer viruses while many others may not have valued or fully understood the invitation, even though we had the collaboration of university professors from the European countries involved. In addition, internet-based research allows those responding to invent realities and there is no safe way of telling the difference between fiction and reality [32]. Notwithstanding, studies carried out over the internet do offer low costs and fast response time as advantages. Another possible limitation is the international nature of the study and consequently the difficulty in analyzing countries with different regulations.

## Conclusions

This study has shown the undervaluation of Ethics Committees, regardless of the country studied. Given its importance, the issue of assisted reproduction should be assessed from an ethical perspective, while addressing matters related to women's health, public health, research, cultural meaning and social impact, as well as economical issues. It is necessary to reinforce the universal principles and values of medical professionalism [33,34]. The principle of respect for the person is vital in bioethics and one of its characteristics is autonomy [7], which could have been more relevant in the professionals' decisions. Hence, major assisted reproduction centers should encourage programs to better prepare professionals in bioethics.

Another aspect made evident by this study is that it is essential that professionals working in assisted reproduction participate more actively in debates and in the drafting of laws and regulations on the use of new technology. The findings in this study suggest that the demands faced daily by MAR professionals are not in accordance with the standing legislation. Italy and Germany, for instance, prove examples of such discrepancy between law and actual practice as MAR is forbidden for single women yet a large number of those interviewed would still perform the procedure. In Brazil, MAR professionals have started to reconsider their stance and have revised the 1992 MAR resolution. The revisited resolution 1957/2010 was finally approved in December 2010 [35].

## Competing interests

The authors declare that they have no competing interests.

## Authors' contributions

SZ, EPP, JRG and DK contributed to the concept and design of the study, data collection, analyses and interpretation of data, and drafting of the manuscript. RF, PGC and BT contributed to data collection and analyses. EC performed the statistical analyses. JRG, JC, AHP, DK and SZ contributed to the concept and design of the study, and critical review of the intellectual content of the manuscript. All authors read and approved the final manuscript.

## Pre-publication history

The pre-publication history for this paper can be accessed here:

http://www.biomedcentral.com/1472-6874/11/21/prepub
